# Long Non-Coding RNA LINC01410 Promoted Tumor Progression *via* the ErbB Signaling Pathway by Targeting STAT5 in Gallbladder Cancer

**DOI:** 10.3389/fonc.2021.659123

**Published:** 2021-07-12

**Authors:** Lili Lu, Shilong Zhang, Zhengqing Song, Weiqi Lu, Zhiming Wang, Yuhong Zhou

**Affiliations:** ^1^ Biotherapy Centre, Zhongshan Hospital, Fudan University, Shanghai, China; ^2^ Liver Cancer Institute, Zhongshan Hospital, Key Laboratory of Carcinogenesis and Cancer Invasion, Ministry of Education, Fudan University, Shanghai, China; ^3^ Department of Medical Oncology, Zhongshan Hospital, Fudan University, Shanghai, China; ^4^ Department of General Surgery, Zhongshan Hospital, Fudan University, Shanghai, China

**Keywords:** long non-coding RNA, LINC01410, STAT5, gallbladder cancer, oncogene

## Abstract

**Objectives:**

Long non-coding RNAs (lncRNAs) have been recently emerging as crucial molecules in multiple human cancers. However, their expression patterns, roles as well as the underlying mechanisms in gallbladder cancer (GBC) remain largely unclear.

**Materials and Methods:**

The expression of lncRNAs in GBC was downloaded from GEO database. Quantitative real-time polymerase chain reaction (qRT-PCR) and RNA *in situ* hybridization (ISH) were used to detect the expression of lncRNAs in GBC tissues. The full-sequence of LINC01410 was determined by RACE assay. Subcellular distribution of LINC01410 was examined by nuclear/cytoplasmic RNA fractionation analysis. Loss- and gain-of-function experiments were conducted to explore the biological functions of LINC01410 *in vitro* and *in vivo*. RNA pull-down, RNA immune-precipitation (RIP), and Western blot assay were conducted to investigate the mechanisms underlying the biological function of LINC01410 in GBC.

**Results:**

LINC01410 was significantly upregulated in the GBC tissues compared to adjacent non-tumor tissues. High LINC01410 expression was significantly associated with poor prognosis of GBC patients. We identified LINC01410 to be 2,877 bp in length and mainly localized in the cytoplasm of GBC cells. Overexpression of LINC01410 promoted GBC cell proliferation, migration, and invasion *in vitro* and GBC progression *in vivo*, whereas LINC01410 downregulation rescued these effects *in vitro*. From RNA pull-down and RIP assay, we identified that STAT5 was a critical downstream target of LINC01410. Furthermore, ErbB signaling pathway was involved in the malignant phenotypes of GBC mediated by LINC01410.

**Conclusions:**

Our results suggested that LINC01410 was an important lncRNA that promoted GBC progression *via* targeting STAT5 and activating ErbB signaling pathway.

## Introduction

Gallbladder cancer (GBC) is the most common biliary tract malignancy and the seventh most common gastrointestinal cancer (1). Despite the encouraging developments and progress in the management of GBC, the 5-year survival of GBC patients remains less than 5% due to its highly aggressive behavior (2–4). Even more unfortunately, early diagnosis is difficult, as there are no specific clinical symptoms or tumor biomarkers for GBC. Therefore, it is of much significance to screen novel and effective targets involved in GBC, which is not only helpful to understand the mechanism of BC tumorigenesis and progression, but also to develop effective anti-tumor drugs.

Long non-coding RNAs (lncRNAs) comprise a wide variety of non-protein coding RNA species with a minimum of 200 nucleotides in length. LncRNAs are primarily thought as since they are unable to encode proteins, and now the lncRNAs have been identified as important actors in the occurrence, development, and progression of multiple malignances, including GBC. For example, it is reported that PVT1 is closely correlated with the poor survival of GBC by regulating miR-143/HK2 axis (5). FOXD2-AS1, which is stabilized by HuR, dismally enhances GBC progression and metastasis through activating miR-502-3p-SET-AKT cascade (6). Despite the growing list of annotated lncRNAs in GBC, the experimentally verified remains small in general. The genome-wide expression patterns, biological roles of lncRNAs in GBC are yet to be elucidated.

In this research, based on bio-informatic analysis of GBC microarray data, we evaluated the expression profiles of lncRNAs in GBC tissues and identified a novel lncRNA, LINC01410 (RefSeq accession number: NR_121647.1), which is highly expressed in GBC. However, its roles in GBC remain unknown. Herein, we aimed to investigate the clinical relevance, biological function, and molecular mechanisms of LINC01410 in GBC progression using bioinformatics and experimental methods.

## Materials and Methods

### Human GBC Tissue Samples

A total of 96 GBC tissues and matched adjacent tissues obtained from Zhongshan Hospital, Fudan University. All tissue specimens were evaluated and approved by the Ethic Committee of Zhongshan Hospital, Fudan University. All patients provided informed consent for inclusion in this study.

### Bioinformatics Analysis

The GBC gene expression data were downloaded from the GEO database. The accession ID was GSE76633, which included nine pairs of GBC tissues and matched adjacent tissues (7). The sample platform used was GPL18180 Agilent-045142 Human LncRNA v4 4X180K.

The microarray files were preprocessed *via* the Robust multichip average method (8, 9). Using the annotation file, the probes were then mapped to the gene symbol. After that, 12,717 unique genes were retained, which included 4,239 protein coding genes and 8,478 lncRNAs based on the biotypes identified using GENECODE v22.

To identify the lncRNAs that are dysregulated in GBC, we used the R package limma to perform the difference analysis and used the pheatmap package to conduct the cluster analysis. The differentially expressed lncRNAs were considered to be significant when |log fold change (FC) | >1.0 and adjusted P-values <0.05.

The GSEA platform (v4.1.0) to perform gene set enrichment analysis (GSEA) on gene signatures associated with ErbB signaling pathway (10) was downloaded from the MSigDB database. The co-expression network between lncRNAs and mRNAs was analyzed in Cor function in the R and visualized by Cytoscape. Gene Ontology (GO) analysis was performed to cluster the lncRNA-related genes by biological functions.

### Cell Lines and Culture

The human GBC cell lines (GBC-SD, SGC-996, G-415, TGBC2TKB, TGBC24TKB, and NOZ) were purchased from the Cell Bank of Chinese Academy of Sciences (Shanghai, China). All cells were maintained in Dulbecco’s modified Eagle’s medium (DMEM) with 10% FBS and 1% penicillin–streptomycin at 37°C in a 5% CO_2_ cell culture incubator.

### RNA Isolation and Quantitative Real-Time PCR

Total RNAs in the frozen tissues and cells were extracted using Trizol reagent (Invitrogen) based on the instructions of the manufacturer. Then the amplification and qRT-PCR were conducted as described previously (11). Primers were as follows: LINC01410, forward: 5′-TCAGAGCCAGGTGACAAGAATG-3′, and reverse: 5′-TGGTTGTCCCTCCTTGTTGCT-3′; *β*-actin, forward: 5′-CACCCAGCACAATGAAGATCAAGAT-3′, and reverse: 5′-CCAGTTTTTAAATCCTGAGTCAAGC-3′.

### Oligonucleotides, Vectors, and Transfections

Small interfering RNAs of LINC01410 (siLINC01410) and scramble siRNAs of LINC01410 mock (siRNA control) were designed and synthesized by GenePharma Co., Ltd (Shanghai, China), and transfected using Lipofectamine 2000 (Thermo Fisher Scientific, MA, USA) based on the instructions of the manufacturer. The sequences of the LINC01410 targeting siRNAs were: LINC01410-homo-1987 (forward: GCUGAUUGAGCAAGAAUUATT, reward: UAAUUCUUGCUCAAUCAGCTT). Sequences of non-target scramble controls were provided by GenePharma (GenePharma, Shanghai, China). CMV-MCS-polyA-EF1A-zsGreen-sv40-puromycin-LINC01410 overexpression lentiviruses and its negative control lentiviruses were infected into GBC-SD cells. Stably expressed clones were selected by qRT-PCR and immunoblotting assays. The primer information were listed as follows: LINC01410(59075-1)-P1: GTGGATCCGAGCTCGGTACCAAAGGGAGAGGGTGAGGGAGTTGTGGAG, LINC01410(59075-1)-P2: ATATTTTATTACCGGTTTAATTAATGCTATTTATACATTTATTGGGTTTGTTAATTATTC.

### Rapid Amplification of cDNA Ends

The 5′- and 3′-RACE assay was conducted using the SMARTer RACE Kit (Clontech, Palo Alto, CA, USA) based on the instructions of the manufacturer. The RACE PCR products were separated on an agarose gel. PCR bands were cloned into pEASY-T1 vector (TransGen Biotech, China) and were sequenced.

### Western Blot Analysis

Cell lysis, electrophoresis, and target protein visualization were performed as described in previous publications (12).

### *In Situ* Hybridization

RNA ISH was conducted to determine the expression level of LINC01410 in the tissues. The slides were deproteinated, hydrated, and deparaffinized, and subsequently pre-hybridized in the hybridization buffer for 1 h at 37°C. Then slides then were hybridized with a double (5′ and 3′) digoxin-labeled LNA probe specific for LOC100133669 (5′-DIG-AGGTCCTGGTTGTCCCTCCTTGTTGCT-3′; Exiqon) at 37°C for overnight. The slides were treated with anti-DIG-HRP and incubated for 40 min at 37°C to develop the brown color. The cells were mounted and observed under a microscopic, and LINC01410 expression was evaluated using ImageJ software.

### Cell Proliferation Assay

Cell proliferation assay was carried out using CCK-8 (Dojin Laboratories, Japan), as described previously (5). Each experiment was independently repeated in triplicate.

### Wound Healing Assay

Cells were placed into six-well plates and incubated for 24 h for the formation of monolayer on the bottom plate. After that, a straight line was scratched onto the monolayer using a 200 μl pipette tip. Next, cells were incubated to allow healing and, the wound was photographed *via* microscopy at 0 and 24 h, respectively.

### Cell Migration Assay

Transwell assays were performed using a 24-well transwell plate (8-μM pore size, Corning, MA, USA) to evaluate the migration and invasion abilities of GBC cells as described before (5). Briefly, the tumor cells in serum-free media were seeded in the upper chamber, and 600 μl complete DMEM medium was added to the lower chamber. The chambers were then maintained at 37°C in 5% CO_2_ for 48 h. Then, cells in the upper surface of the filters were gently wiped away using a cotton swab. The invasive tumor cells from the upper surface were fixed in 4% paraformaldehyde and stained with hematoxylin. Each experiment was independently repeated in triplicate.

### Nuclear/Cytoplasmic RNA Fractionation Analysis

Approximately 1.0 × 10^7^ GBC-SD cells were collected in order to determine the cellular localization of LINC01410. Nuclear and cytoplasmic RNAs were extracted and purified using the Cytoplasmic & Nuclear RNA Purification Kit (Norgen, Belmont, CA) according to the instructions of the manufacturer. The detailed methods used were described previously (11).

### RNA Pull-Down Assay

LINC01410 full-length sense, antisense, and serial deletion sequences were transcribed with Biotin RNA Labeling Mix and T7 RNA polymerase (Roche, Switzerland), and purified with the RNeasy Mini Kit (Qiagen, USA) as described by the manufacturers. Biotin labeled LINC01410 was incubated with total cell lysates of GBC, and eluted proteins were purified and detected by Western blot assay.

### RNA Immunoprecipitation Assay

We performed RIP assays using the Magna RIP RNA-Binding Protein Immunoprecipitation Kit (Millipore, Bedford, MA, USA). Briefly, cells were lysated in lysis buffer containing protease inhibitor cocktail and RNase inhibitor. Then, cell extracts were incubated with magnetic beads conjugated with control IgG or anti-STAT5 antibody. After washing, Western blot was conducted to quantify the immunoprecipitation of RNAs.

### Animal Models for Tumor Growth and Metastasis

The animal studies were strictly conducted in accordance with the protocols approved by the Ethic Committee of Zhongshan Hospital, Fudan University. Briefly, 2.0 × 10^6^ GBC-SD cells with vector or OE-LINC01410 stable transfection were subcutaneously injected into the left flanks of nude Balb/c mice. The mice were euthanized, and the tumors were collected and weighed after 37 days. For metastasis *in vivo*, LINC01410 over-expressing GBC-SD cells were harvested and re-suspended. Hepatic and pulmonary metastases models were established by intrasplenic injection of 2.0 × 10^6^ tumor cells and splenectomy according to prior publications (13). The mice were euthanized and the metastatic tissues were collected after 6 weeks.

### Statistical Analysis

Statistical analysis was carried out using R, or GraphPad Prism 7.0 software. Two-tailed Student’s t-test, Chi-square test, or log-rank test was used for the indicated comparisons. *p* < 0.05 was considered to be statistically significant (* *p* < 0.05, ** *p* < 0.01, *** *p* < 0.001, ****p < 0.0001, ns, no significance).

## Results

### Distinct Expression Profiles of lncRNAs Between Paired GBC and Non-Tumor Samples

**Figure 1:**
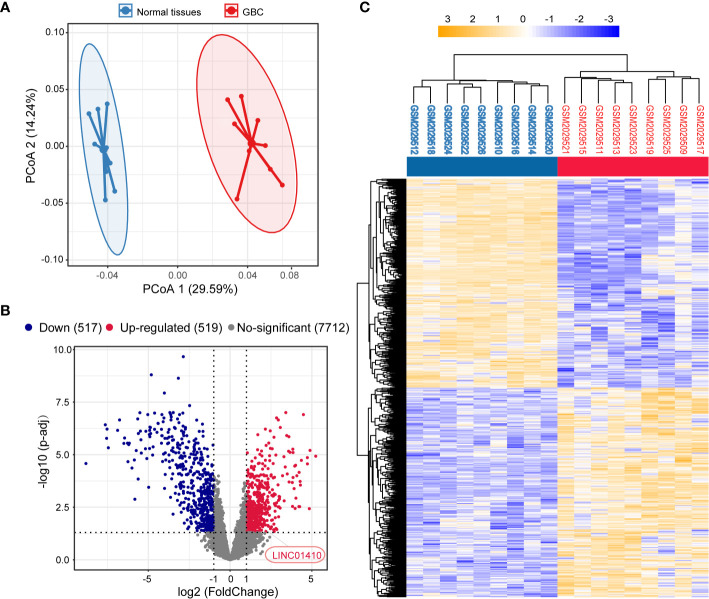
Identification of differentially expressed lncRNAs between the GBC and matched non-tumor samples. **(A)** Principal component analysis (PCA) of the lncRNA expression profiles in each sample. **(B)** Volcano plot of the differentially expressed lncRNAs. **(C)** Two-dimensional diagram of differential expressions of 1,036 lncRNAs, and the data were organized by transcript and tumor and non-tumor category based on similarity.

First, the GEO dataset GSE76633 was used to investigate the lncRNA expression in GBC, which included nine pairs of GBC and corresponding non-tumor samples. PCoA analysis revealed distinct expression profiles of lncRNAs between GBC and paired non-tumor samples **(****Figure 1A****)**. A total of 1,036 significantly differentially expressed lncRNAs were identified by DESeq2, among which 519 were upregulated in GBC samples, and 517 were downregulated **(** **Figure 1B****)**. The clustering heatmaps were shown in **Figure 1C**.

### LINC01410 Was Upregulated in GBC and Associated With GBC Poor Survival

**Figure 2:**
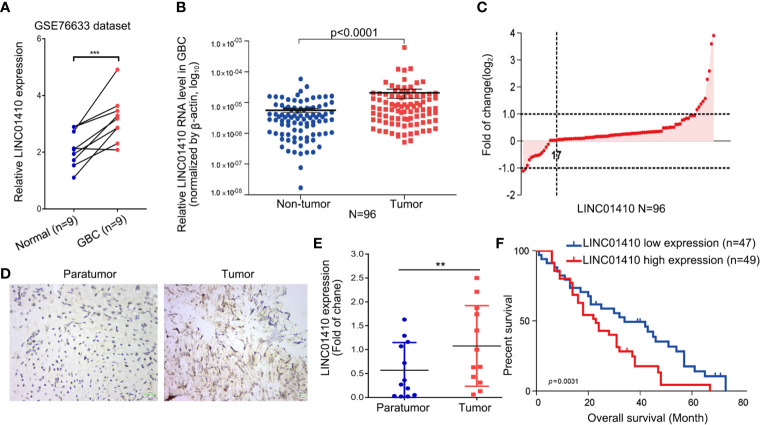
LINC01410 was significantly upregulated in GBC tissues and cells and associated with poor prognosis. **(A)** LINC01410 expression levels in GBC tissues and paired non-tumor tissues in the GSE76633 dataset. ***p < 0.001. **(B)** LINC01410 expression in GBC tissues detected by qRT-PCR in 96 pairs of GBC tissues and paired non-tumor tissues. **(C)** Distribution of LINC01410 expression in 96 pairs of GBC tissues. **(D)** Representative images of ISH for LINC01410 expression in GBC tissues and paired non-tumor tissues. **(E)** ISH analysis of LINC01410 expression in the GBC tissues and paired non-tumor tissues (n = 12). **p < 0.01. **(F)** Kaplan–Meier analysis of the overall survival of GBC patients stratified by LINC01410 expression (n = 96).

Among these differentially expressed lncRNAs, lncRNA-LINC01410 (NR_121647.1), which had never been investigated in GBC, was selected as the GBC-associated lncRNA. LINC01410 was highly expressed in GBC compared to non-tumor samples **(** **Figure 2A** **)**. Furthermore, we detected LINC01410 expression in 96 pairs of GBC samples and adjacent non-tumor tissues using qRT-PCR and found that LINC01410 was more highly expressed in CRC tissues than in the paired non-tumor tissues **(****Figure 2B** **)**, and LINC01410 was over-expressed more than 2.0 fold in 82.3% of GBC samples **(****Figure 2C****)**. Furthermore, we collected tissues from 12 GBC patients and used ISH to investigate the expression of LINC01410 **(****Figure 2D****)**. Also, LINC01410 is significantly highly expressed in GBC tumors compared with normal tissues **(****Figure 2E****)**. Next, to investigate the association between LINC01410 expression and the clinicopathological characteristics of GBC patients, the 96 GBC patients were classified into two groups according to the median of LINC01410 expression. TheChi-square test showed that LINC01410 expression was clearly associated with pathological stage and lymph node invasion in GBC patients **(****Table 1****)**. Moreover, we investigated the association between LINC01410 expression and prognosis in GBC patients. Kaplan–Meier survival analysis showed that high expression of LINC01410 was associated with poor overall survival **(****Figure 2F****)**. Collectively, these results indicated that LINC01410 can represent an enhancer in the development and progression of GBC.

**Table 1:** The relationship between LINC01410 expression and clinicopathologic characteristics of GBC patients in the present study.

Characteristic	Cases	LINC01410 expression		χ^2^ value	*P*-value
		Low (%)	High (%)		
**Gender**				0.133	0.715
Male	34	18 (38.3)	16 (32.7)		
Female	62	29 (61.7)	33 (67.3)		
**Age**				2.037	0.154
<60	35	21 (44.7)	14 (28.6)		
≥60	61	26 (55.3)	35 (71.4)		
**Tumor site**				4.707	0.963
Gallbladder neck	34	16 (34.0)	18 (36.7)		
Gallbladder body	22	11 (23.4)	11 (22.4)		
Gallbladder fundus	40	20 (42.6)	20 (40.9)		
**Tumor size**				10.672	0.001
≤3 cm	42	29 (61.7)	13 (26.5)		
>3 cm	54	18 (38.3)	36 (73.5)		
**Pathological stage**				24.666	<0.001
I–II	38	31 (66.0)	7 (14.3)		
III–IV	58	16 (34.0)	42 (85.7)		
**Vascular invasion**				0	1
No	43	21 (44.7)	22 (44.9)		
Yes	53	26 (55.3)	27 (55.1)		
**Lymph node invasion**				16.633	<0.001
No	46	33 (70.2)	13 (26.5)		
Yes	50	14 (29.8)	36 (73.5)		

### Basic Characterization of LINC01410

**Figure 3:**
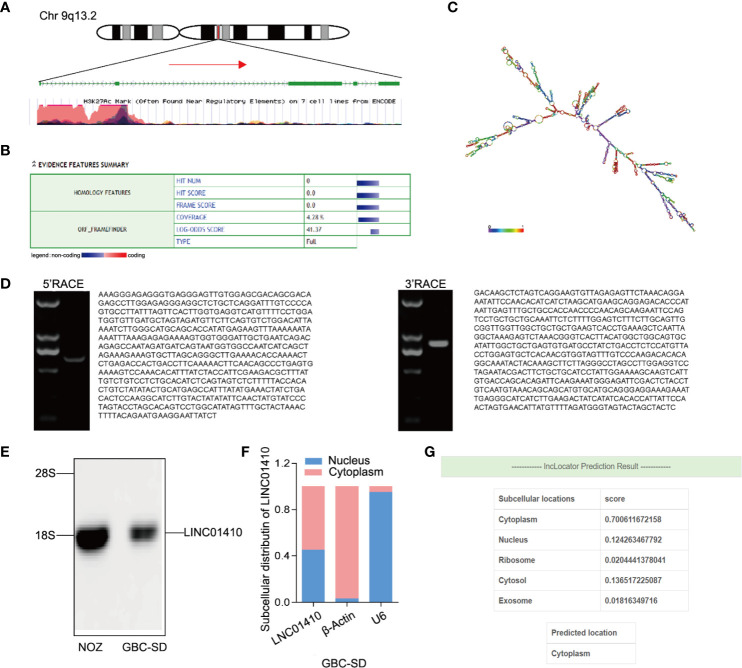
Basic characterization of LINC01410. **(A)** Schematic diagram of LINC01410. **(B, C)** ORF finder **(B)** and secondary structure **(C)** prediction for protein-coding potential of LINC01410. **(D)** Gel electrophoresis of nested PCR products from 5′-RACE and 3′-RACE. **(E)** Western blot analysis of LINC01410 transcripts in NOZ and GBC-SD cell lines. **(F)** Subcellular localization of LINC01410 in GBC cells using cell fractionation assays. **(G)** The location of URRCC predicted by lncLocator.

Using UCSC Genome Browser, we further confirmed that LINC01410 was located on the human chromosome 9: q13.2 **(** **Figure 3A** **)**. Analysis of the sequences using the Open Reading Frame Finder and codon substitution frequency (CSF) analysis indicated that LINC01410 had weak potential to code proteins **(** **Figures 3B, C** **)**. RACE analysis resulted in the identification of full sequence of LINC01410, with a transcript length of 2,877 bp **(** **Figure 3D** **)**. In addition, the existence and size of LINC01410 were confirmed using northern blot assays **(** **Figure 3E** **)**. Furthermore, we analyzed the subcellular localization of LINC01410 in GBC cells using cell fractionation assays and found that LINC01410 was mainly located in the cytoplasm of GBC cells **(** **Figure 3F** **)**, which was consistent with online software lncLocator predicting the localization of LINC01410 **(** **Figure 3G** **)**.

### LncRNA-mRNA Co-Expression Network Revealed the Potential Role of LINC01410 in GBC

**Figure 4:**
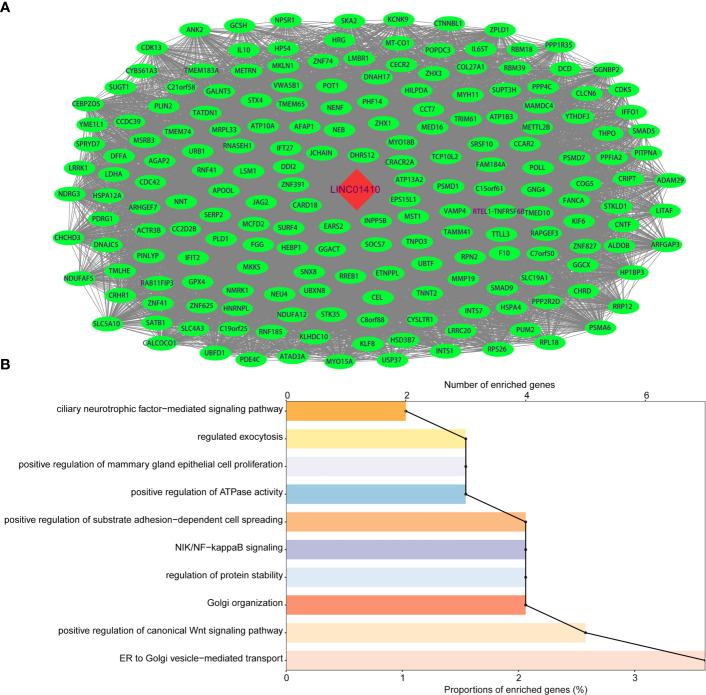
Functional enrichment analysis of protein-coding genes co-expressed with LINC01410. **(A)** Network of protein-coding genes co-expressed with LINC01410 in GBC. Red nodes represented LINC01410, and green nodes represented LINC01410-correlated PCGs. **(B)** GO enrichment terms significantly associated with protein-coding genes in GBC.

LncRNA itself does not encode proteins, but its functions are known to be closely associated with the biological processes in which co-expressed protein-coding genes (PCGs) are also potentially involved (14, 15). Therefore, we performed Spearman’s correlation for paired LINC01410 and PCG expression in the GBC patients from GSE76633 dataset. Using |coefficient| >0.6 and *p <*0.05 as the cut-off criterion, a total of 195 mRNAs were considered as LINC01410-correlated PCGs, and then construct co-expression networks **(** **Figure 4A** **)**.

To explore potential functions of LINC01410 in GBC, we performed GO enrichment analysis of LINC01410-correlated PCGs. The results revealed that these PCGs were significantly enriched in positive regulation of substrate adhesion-dependent cell spreading, NIK/NF-kappaB signaling, and positive regulation of canonical Wnt signaling pathway **(** **Figure 4B** **)**. Interestingly, these enriched GO functions have been reported to be associated with GBC through literature investigation. Hence, LINC01410 can participate in GBC tumorigenesis *via* regulating the PCGs to influence several known GBC-associated biological functions.

### LINC01410 Promoted GBC Cell Proliferation, Migration, and Invasion *In Vitro*

**Figure 5:**
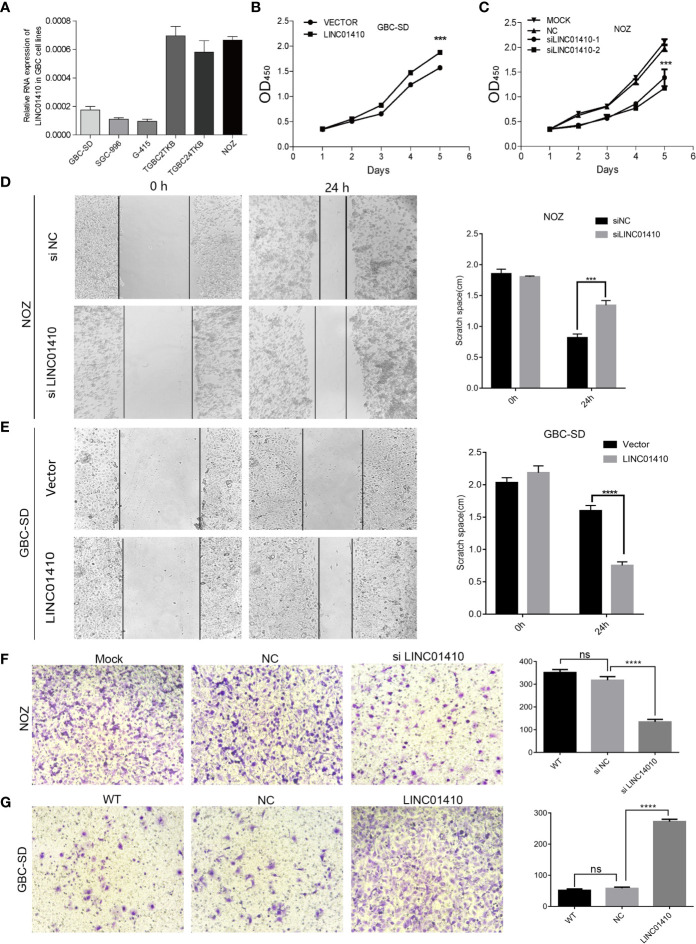
LINC01410 promoted the development and progression of GBC in *vitro*. **(A)** qRT-PCR assay for LINC01410 expression levels in indicated GBC cell lines. **(B, C)** The effects of LINC01410 on cell proliferation were determined using CCK-8 assay. **(D, E)** The effects of LINC01410 on cell migration capability by wound healing assay. **(F, G)** Transwell assays were performed to determine the role of LINC01410 in the migration of GBC cells. Representative images of cell migration and statistical analysis of specific migrated cell number were shown. Scale bar, 100 μm. ***p < 0.001; ****p < 0.0001; ns, not significance.

To confirm whether LINC01410 was involved in GBC tumorigenesis, we conducted loss- or gain-of-function experiments in GBC cells. First, we measured the LINC01410 expression in six human GBC cell lines using qRT-PCR (**Figure 5A**). Next, we selected the NOZ cells with relatively high expression of LINC01410 and the GBC-SD cells with relatively low expression for subsequent experiments. NOZ cells were transfected with siRNA to reduce LINC01410 expression, while GBC-SD cells were transfected with overexpression lentiviruses to establish the cells stably expressing LINC01410. Using qRT-PCR, we confirmed that knockdown and overexpression efficiency of LINC01410 in NOZ cells **(** **Supplementary Figure S1** **)** and GBC-SD cells **(** **Supplementary Figure S2** **)**, respectively. CCK8 assays showed that overexpression of LINC01410 promoted GBC cell proliferation in GBC-SD cells, whereas downregulation of LINC01410 in NOZ cells suppressed the cell proliferation compared with a negative control group **(** **Figures 5B, C** **)**. Thus, the data revealed that LINC01410 promoted GBC cell proliferation *in vitro*.

Subsequently, we investigated the potential of LINC01410 in regulating GBC cell migration and invasion. Wound healing assays indicated that downregulation of LINC01410 significantly suppressed the migration capability of NOZ cells **(** **Figure 5D** **)**. Furthermore, transwell assay also revealed that the invasion of NOZ cells was blocked after downregulation of LINC01410 **(** **Figure 5F** **)**. Conversely, the migration **(** **Figure 5E** **)**, as well as invasion ability **(** **Figure 5G** **)** of GBC-SD cells was obviously enhanced after overexpression of LINC01410. Collectively, LINC01410 functioned as an oncogene through promoting GBC cell proliferation, migration, and invasion *in vitro*.

### LINC01410 Promoted GBC Progression *In Vivo*

**Figure 6:**
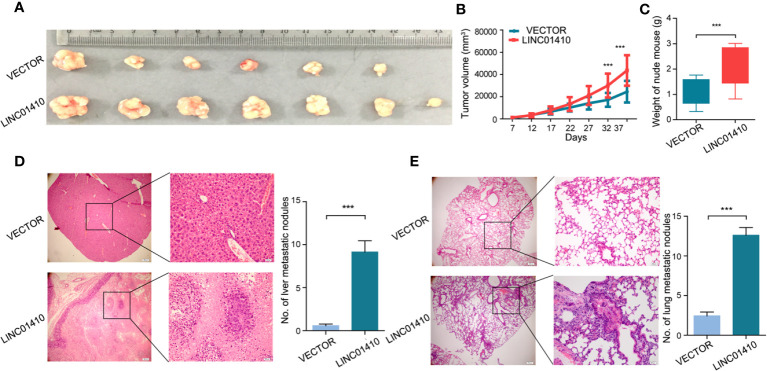
LINC01410 enhanced the development and progression of GBC *in vivo*. **(A–C)** The GBC-SD cells with LINC01410-overexpressed lentiviruses and its negative control lentiviruses were injected subcutaneously into nude mice, respectively. The established tumors **(A)**, tumor growth curves **(B)** and tumor weights **(C)** were shown (n = 6). **(D, E)** LINC01410 over-expressing cells were injected into the spleen of nude mice. Representative microscopic images, and number of liver **(D)**, and lung **(E)** metastatic lesions at 6 weeks after injection of tumor cells. ***p < 0.001.

To explore the oncogenic role of LINC01410 on GBC *in vivo*, the GBC-SD cells with LINC01410-overexpressed lentiviruses and its negative control lentiviruses were injected subcutaneously into nude mice, respectively. As **Figures 6A, B** show, overexpression of LINC01410 led to dramatically fast tumor growth. This result was confirmed by tumor weights from each group (**Figure 6C**). Metastasis to distant organs is a dismal feature of GBC, and more than 50% of GBC patients would develop distant metastases (16, 17). To investigate the role of LINC01410 on GBC metastasis, the GBC-SD cells with LINC01410-overexpressed lentivirus were injected into the spleen. After 6 weeks, the mice were euthanized and the metastatic lesions in liver and lung were counted. Elevated LINC01410 expression led to increased numbers of metastasis to liver **(** **Figure 6D** **)** and lung **(** **Figure 6E** **)**. Taken together, the oncogenic role of LINC01410 in GBC was confirmed *in vivo*.

### STAT5 Is a Crucial Downstream Target of LINC01410

**Figure 7:**
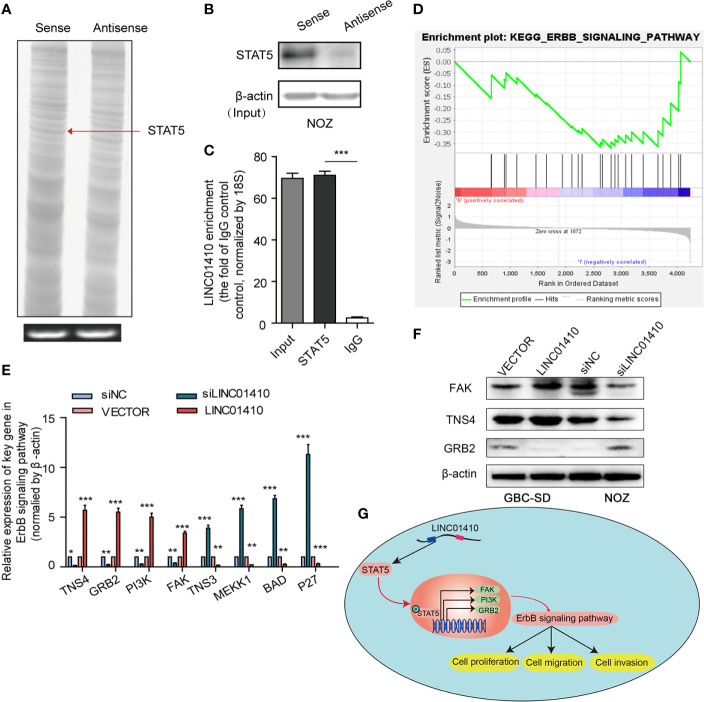
STAT5 is a key downstream target of LINC01410. **(A)** Sliver staining of LINC01410-correlated proteins following RNA pull-down assay. STAT5, included in the differential band indicated by the red arrow, was identified as one of the LINC01410-correlated proteins using mass spectrometry analysis. **(B)** Western blot was performed to validate this interaction using anti-STAT5 antibody. *β*-actin was used as a negative RNA control. **(C)** RIP assay was performed to recover the interaction of LINC01410 and STAT5. **(E)** GSEA analysis of ErbB signaling pathway signatures in the GSE76633 dataset. **(E, F)** The mRNA **(E)**, and protein levels **(F)** of target genes involved in the ErbB signaling pathway detected by RT-PCR and Western blot, respectively. **(G)** Schematic model showing LINC01410-based signaling pathway in GBC cells proliferation, migration, and invasion. *p < 0.05; **p < 0.01; ***p < 0.001.

Our next aim was to investigate the mechanisms through which LINC01410 functioned its oncogenic role in GBC. The LncRNA has been demonstrated to interact with PCGs to exert its biological functions (18, 19). Given the lncRNA–mRNA co-expression network constructed above, we speculated that LINC01410 can function through the same way. To search for the PCGs that interacted with LINC01410, we performed RNA pull-down assay **(** **Figure 6A** **)**. Differential bands between LINC01410 sense and antisense RNA were extracted for subsequent mass spectrometry. One of the LINC01410-correlated proteins was identified as signal transducer and activator of transcription 5 (STAT5) **(** **Figure 7A** **)**. The interaction between LINC01410 and STAT5 was also validated by Western blot assay **(** **Figure 7B** **)**. To investigate how LINC01410 regulated the expression of STAT5 protein, we performed RIP assays, and the results demonstrated that STAT5 protein strongly enriched LINC01410 in NOZ cells, confirming the specific target relationship between LINC01410 and STAT5 **(** **Figure 7C** **)**. Collectively, STAT5 was regulated by LINC01410 and can function as a downstream target of LINC01410.

### LINC01410 Is Associated With ErbB Signaling Pathway

STAT5 is a multifunctional transcriptional factor known to be involved in cell growth, differentiation, proliferation, and apoptosis (20, 21). Studies have demonstrated that STAT5 can result in stimulation of ErbB signaling pathway (22, 23), whose aberrant status played an important role in GBC (24). To investigate whether ErbB signaling pathway was involved in the malignant phenotypes mediated by LINC01410, we performed GSEA analysis of the genes between GBC tissues with high LINC01410 expression and low LINC01410 expression in the GSE76633 dataset **​(** **Figure 7D** **)**. Interestingly, high expression of LINC01410 was positively correlated with ErbB signaling pathway, indicating LINC01410 can potentially regulate ErbB signaling pathway in GBC. Hence, we detected the expression of the target genes involved in the ErbB signaling pathway in both LINC01410-knockdown GBC-SD cells and LINC01410-over-expression ONZ cells. We observed that the expression of TNS4, GRB2, PI3K, and FAK was significantly decreased after LINC01410 knockdown, but increased after LINC01410 overexpression **(** **Figure 7E** **)**. These results were further confirmed by Western blot **(** **Figure 7F** **)**. Taken together, these data suggested that LINC01410 positively regulated ErbB signaling pathway, which can lead to the malignant phenotypes of GBC **(** **Figure 7G** **)**.

## Discussion

With the development of high-throughput sequencing and bioinformatics, numerous lncRNAs have recently been identified from the transcription of human genome (25). LncRNAs have been emerging in various biological processes and diseases, particularly in malignant tumors (26–28). For example, lncRNA LINC00673 inhibits tumor progression *via* reinforcing the interaction of PTPN11 with PRPF19 and facilitated STAT1-dependent antitumor response (29). LncRNA URRCC facilitates proliferation and metastasis of renal cancer by regulating the EGFL7/P-AKT/FOXO3 signaling pathway and indicates poor survival in patients (30). However, most of the lncRNAs involved in GBC have yet to be investigated. GBC is one of the most aggressive malignant tumors with prevalence rate rising in recent years (1). Hence, it was necessary to explore the biological basis of lncRNAs underlying GBC development and progression and identify novel targets for diagnosis and treatment.

In this study, we observed distinct expression profiles of lncRNAs between paired GBC and non-tumor samples, and screened the differentially expressed lncRNAs by gene microarray. We focused our attention on a functionally unknown lncRNA in GBC, LINC01410. In the NCBI database (https://www.ncbi.nlm.nih.gov/gene/103352539), we found that LOC100132707 was widely expressed in human tissues, such as appendix, bone marrow, spleen, gall and bladder. Recent studies have reported that LINC01410 is overexpressed in several cancers, including colon tumor (31), gastric cancer (32), and thyroid carcinoma (33). However, its role and potential mechanisms involved in GBC have been unexplored. In the present study, we evidenced that LINC01410 was significantly upregulated in GBC cells and tissues and closely associated with poor survival of GBC patients, indicating LINC01410 was potentially involved in the progression of GBC. These results prompted us to investigate LINC01410 in GBC more thoroughly. To do this, we defined the full-length of LINC01410 transcript in GBC cells using RACE assay for the first time.

To explore its function in GBC, we then carried out gain- and loss-of-function experiments in CBG cells. The results showed that overexpression of LINC01410 significantly promoted GBC cell proliferation, migration, and invasion capabilities *in vitro*, while knockdown of LINC01410 inhibited cell proliferation, migration, and invasion. Further experiments also showed that overexpression of LINC01410 also induced a significant acceleration in the tumor growth *in vivo*, together with increased rate of metastasis to distant organs. To our knowledge, this was the first study to report the biological roles of LINC01410 in GBC.

Several studies have suggested that the biological role of lncRNAs is usually related to their unique subcellular localizations (34). Cell fractionation assays showed that LINC01410 was mainly localized in the cytoplasm, which was consistent with the prediction from lncLocator, indicating that LINC01410 was likely to interact with molecules within the cytoplasm. The cytoplasmic lncRNAs are known to serve as mRNA decoys to influence mRNA translation or stability (35). In RNA pull-down and RIP assay, we identified that STAT5 was a critical downstream target of LINC01410. STAT5 belongs to one of the members of signal transducer and activator of transcription (STAT) family (36). It has two highly homologous isoforms, STAT5A and STAT5B. Compared with other members, STAT5 participates in a wider range of cell physiological regulation (37). STAT5 is proven as a multifunctional transcription factor involved in various processes (38, 39) and also as a tumor accelerator in multiple malignancies including GBC since persistent activation of STAT5 is the chief culprit of tumorigenesis (40, 41). Therefore, targeting STAT5 is a potential anticancer strategy (42, 43). Hence, we demonstrated that INC01410 expressed in the cytoplasm of GBC cells directly binds to STAT5, thereby regulating the malignant biological behaviors of GBC.

STAT5 is an important transcription factor in ErbB signaling pathway, which is involved in the regulation of cell proliferation, migration, differentiation, apoptosis (44). Maolan et al. (24) analyzed the exon sequencing data of 57 pairs of GBC and paired adjacent cancer samples and found that the ErbB signaling pathway was the most significant pathway with mutation enrichment, and the aberration of ErbB signaling pathway was associated with worse survival of GBC patients. The results indicated that the ErbB signaling pathway might play a crucial role in the pathological process of GBC. However, the roles of ErbB signaling pathway involved in GBC had not been reported. In our study, we found that LINC01410 expression was positively correlated with the ErbB signaling pathway in the GBC using bioinformatics analysis. Furthermore, gain- and loss-of-function experiments showed that LINC01410 considerably activated ErbB signaling pathway in GBC cells, which contributed to LINC01410-mediated malignant phenotypes of GBC

However, there were several limitations to this study, such as the limited size of cohort and detailed clinicopathological information of GBC patients. The potential mechanisms for LINC01410 overexpression in GBC also remained unclear and the exact interaction between LINC01410 and STAT5 should be elucidated in detail. Another limitation was that we did not clarify the effects of ErbB signaling pathway on LINC01410-mediated promotion in tumor development and progression. We planned to address the above problems in a future study.

In summary, LINC01410 expression was overexpressed and associated with poor survival in GBC patients. LINC01410 promoted GBC progression *in vitro* and *in vivo*. Mechanistically, LINC01410 promoted GBC progression by regulating STAT5 expression and activating ErbB signaling pathway. Thus, our findings identified LINC01410 as a novel oncogene in GBC, which is a potential promising target for diagnosis and therapy in GBC.

## Data Availability Statement

The raw data supporting the conclusions of this article will be made available by the authors, without undue reservation.

## Ethics Statement

The studies involving human participants were reviewed and approved by the Ethic Committee of Zhongshan Hospital, Fudan University. The patients/participants provided their written informed consent to participate in this study. The animal study was reviewed and approved by the Ethic Committee of Zhongshan Hospital, Fudan University.

## Author Contributions

YZ and ZW designed and finalized the study. SZ conducted bioinformatics analysis. LL and ZS performed the experiments. WL helped on samples collection and experiments. YZ and SZ drew the charts and wrote the paper. All authors contributed to the article and approved the submitted version.

## Funding

The present study was supported by grants from Shanghai Science and Technology Committee Project (No. 18ZR1406200).

## Conflict of Interest

The authors declare that the research was conducted in the absence of any commercial or financial relationships that could be construed as a potential conflict of interest.

## Supplementary Material

Supplementary Figure 1 The expression of LINC01410 was determined by qRT-PCR in NOZ cells after transfection with the siRNA or siRNA-NC.

Supplementary Figure 2 The expression of LINC01410 was determined by qRT-PCR in GBC-SD cells after transfection with the LINC01410 overexpression lentiviruses and its negative control lentiviruses.
