# Supplementary figures and images for: Long Non-Coding RNA LINC01410 Promoted Tumor Progression via the ErbB Signaling Pathway by Targeting STAT5 in Gallbladder Cancer
Source: Front Oncol. 2021 Jul 12;11:659123. doi: 10.3389/fonc.2021.659123 (PMC8312242; doi:10.3389/fonc.2021.659123)

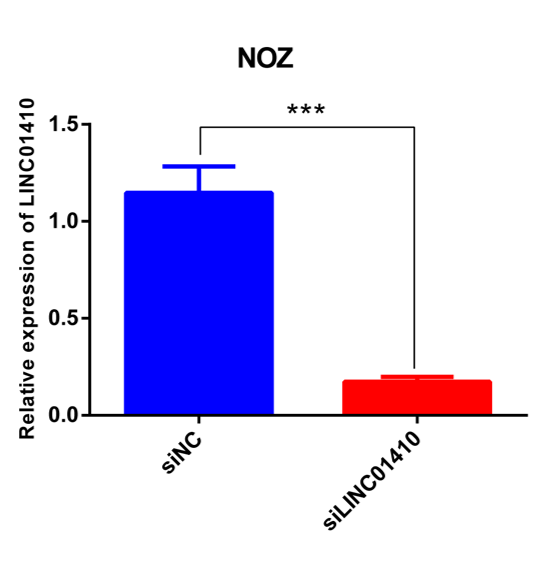

Supplement: Supplementary Figure 1 — The expression of LINC01410 was determined by qRT-PCR in NOZ cells after transfection with the siRNA or siRNA-NC. [file Image_1.tif]

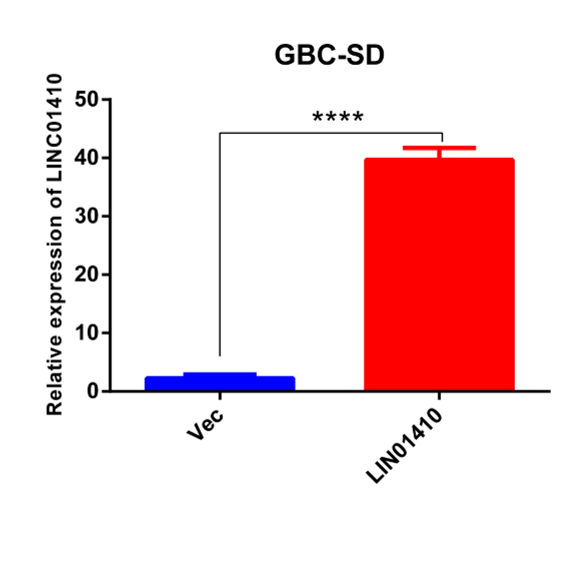

Supplement: Supplementary Figure 2 — The expression of LINC01410 was determined by qRT-PCR in GBC-SD cells after transfection with the LINC01410 overexpression lentiviruses and its negative control lentiviruses. [file Image_2.tif]
